# Duration of Cord Clamping and Neonatal Outcomes in Very Preterm Infants

**DOI:** 10.1371/journal.pone.0138829

**Published:** 2015-09-21

**Authors:** Dongli Song, Priya Jegatheesan, Glenn DeSandre, Balaji Govindaswami

**Affiliations:** Department of Pediatrics, Division of Neonatology, Santa Clara Valley Medical Center, San Jose, California, United States of America; NIH, UNITED STATES

## Abstract

**Background:**

Delayed cord clamping (DCC, ≥30s) increases blood volume in newborns and is associated with fewer blood transfusions and short-term neonatal complications. The optimal timing of cord clamping for very preterm infants should maximize placental transfusion without interfering with stabilization and resuscitation.

**Aim:**

We compared the effect of different durations of DCC, 30-45s vs. 60-75s, on delivery room (DR) and neonatal outcomes in preterm infants <32 weeks gestational age (GA).

**Methods:**

This is a single-center prospective observational study. Data were collected prospectively from eligible infants from two groups: 30-45s DCC group (January 2008 to February 2011, n = 187) and 60-75s DCC group (March 2011 to April 2014, n = 166).

**Results:**

The 60-75s DCC group compared to the 30-45s DCC group had higher hematocrits at <2 hours (49.2% vs. 47.4%, p = 0.02). In infants <28 weeks GA, the 12–36 hours hematocrit was higher in the 60-75s DCC group compared to the 30-45s DCC group (47.9% vs. 42.1%, p = 0.002). The 60-75s DCC group had reductions in DR intubation (11% vs. 22%, p = 0.004), hypothermia on admission (1% vs. 5%, p = 0.01), surfactant therapy (13% vs. 28%, p = 0.001), intubation in the first 24 hours (20% vs. 34%, p = 0.004), any intubation (27% vs. 40%, p = 0.007), and any red blood cell transfusion (20% vs. 33%, p = 0.008) during the hospitalization compared to the 30-45s DCC group. These reductions remained significant after adjusting for GA, gender and >48 hours of antenatal steroid exposure. There was no difference between the two groups in neonatal death, intraventricular hemorrhage, chronic lung disease, late onset sepsis, necrotizing enterocolitis and severe retinopathy of prematurity.

**Conclusion:**

In this study cohort increasing DCC duration from 30-45s to 60-75s is associated with decreased hypothermia on admission, neonatal respiratory interventions and red blood cell transfusions without increase in neonatal mortality and morbidities.

## Introduction

Placental transfusion at birth is a physiological process that optimizes the immediate transition to extra-uterine life [1]. Delayed cord clamping (DCC, ≥30s) allows for larger placental transfusion and increases blood volume in newborns [2–4]. In a recent meta-analysis [5], DCC in preterm infants was associated with less need for blood transfusion and reduced risk of intraventricular hemorrhage (IVH) and necrotizing enterocolitis (NEC). Randomized clinical trials have shown other benefits of DCC in preterm infants including improved cardiovascular stability [6–11], cerebral oxygenation [7], and lower risks for both severe IVH and late onset sepsis (LOS) [12]. In recent years DCC has been recommended by the World Health Organization [13, 14], the Neonatal Resuscitation Program (NRP) [15] for at least 60 seconds in term and preterm infants not requiring resuscitation and by the American Congress of Obstetricians and Gynecologists [16] for 30 to 60 seconds in preterm infants not requiring resuscitation.

Effective placental transfusion depends on multiple factors including the timing of cord clamping, position of the infant relative to the placenta, the infant’s respiratory effort and uterine contraction. Very premature infants require stabilization and are at a higher risk for requiring resuscitation at birth. The optimal timing and method of cord clamping for very preterm infants needs to balance the benefits of DCC for larger placental transfusion and the risks of interfering with initiation of stabilization and resuscitation. Studies conducted in the preterm population have compared outcomes of immediate cord clamping (ICC), where the cord was clamped within a few seconds of birth, with DCC of 30-180s [5] or with cord milking [17–19]. The effects of different durations of DCC on the delivery room (DR) and neonatal outcomes remain to be studied.

In January 2008, we implemented 30-45s of DCC as part of a standardized DR management of very preterm infant delivery. In March 2011, we extended the duration of DCC to 60-75s. The objective of this study was to compare the impact of the two different durations of DCC on the DR and neonatal outcomes of very preterm infants.

## Methods

This observational study was conducted from January 2008 to April 2014 at the Santa Clara Valley Medical Center (SCVMC), a safety-net teaching hospital with a high-risk obstetric service and a regional level III Neonatal Intensive Care Unit (NICU). Data was collected prospectively from January 2008. This study was approved by the SCVMC institutional review board as a quality improvement project and informed consent was waived.

In 2007 we developed a standardized, scripted approach to DR management of very preterm infants born at < 32 weeks gestation called Smart Start which included 30-45s DCC [20]. Prior to the implementation of DCC, the standard of care was ICC approximately 5-10s after delivery. We began 30-45s of DCC in July 2007 and fully implemented all elements of the Smart Start bundle by January 2008. In March 2011, we extended the duration of DCC to 60-75s after evaluation of our safety data and a new NRP recommendation advocating 1 minute DCC for infants not requiring resuscitation [15]. The percent of infants who received DCC was similar between the two study periods (78% in 30-45s DCC group and 81% in 60-75s DCC group). Our NICU criteria for DR resuscitation, intubation, surfactant therapy and blood transfusion as well as the range of targeted oxygen saturation in preterm infants were not changed during the study period.

Our DCC protocol was developed based on published methods [12, 21]. Prior to delivery, pediatric and obstetric providers jointly determine the infant’s eligibility for DCC. Contraindications for DCC included obstetric (placental or cord causes, i.e. placental abruption, placenta previa, cord avulsion and true knot) and fetal or neonatal causes (i.e. severely compromised infant without spontaneous respiration requiring immediate resuscitation after birth). After delivery, the preterm infant was held by a pediatric provider in a polyethylene wrap (vaginal births) or a sterile towel (Cesarean Section, CS) overlying a chemical warming mattress. The infant was held as low as possible, without creating tension on the cord, below the level of the mother’s introitus at vaginal delivery or below the level of incision at CS. After the infant was delivered, a second pediatric provider verbalized the time in 5-10s intervals. Intermittent tactile stimulation by gentle rubbing on the back and oral bulb suctioning were performed prior to cord clamping but neither positive pressure ventilation nor cord milking was done. Once the intended duration of DCC was achieved, the pediatric provider called for cord clamping. Cord was cut before intended DCC duration if the infant was not able to maintain respirations with stimulation, as determined by the pediatrician, or due to placental separation or bleeding, as determined by the obstetrician. If necessary positive airway pressure, intubation and chest compression were initiated after the cord was cut and the infant was placed on an open warmer. Apgars were assigned from the time of birth, when the baby is delivered and prior to cord clamping. In both vaginal and CS deliveries, mother received a dose of Oxytocin after delivery of the infant and after the cord was clamped.

We collected demographics, cord clamping data and neonatal outcomes at discharge prospectively from infants who received DCC. Data were collected from two groups of infants who received the intended duration of DCC: the 30-45s DCC group born between January 2008 and February 2011, and the 60-75s DCC group born between March 2011 and February 2014. We compared demographic variables, hematocrits, DR measures and NICU measures as well as neonatal mortality and morbidity between the two groups. Demographic variables included gestational age (GA), birth weight, gender, antenatal steroids (any and >48 hour exposure) and CS. Hematocrits were obtained when clinically indicated. Hematocrit data included in this analysis were those collected at <2 hours (<2h, median time 0.8h) and 12–36 hours (12-36h, median time 19.5h). Hematocrits obtained after blood transfusion were excluded. DR measures included Apgars at 1 and 5 minutes, intubation, chest compressions, resuscitation medications and temperature on NICU admission. NICU measures included surfactant administration, intubation within the first 24 hours of life, intubation and red cell transfusion at any time during the NICU stay. Neonatal morbidity included IVH, late onset sepsis (LOS), NEC, chronic lung disease (CLD), and severe retinopathy of prematurity (ROP) as defined as >stage 2 or with Plus disease or requiring surgery or anti-vascular endothelial growth factor treatment for ROP.

We performed t-tests to compare normally distributed variables, the Mann-Whitney rank-sum test for non-normally distributed continuous variables and chi-square or Fisher’s exact test to compare categorical variables. Multivariate logistic regression analysis was done for outcomes that were significant between the groups to adjust for GA, gender and antenatal steroids >48 hours based on face validity of their effect on outcomes. Statistical analyses were performed using statistical data analysis software, STATA 10.0 (Statacorp, College Station, Texas). P value of <0.05 was considered significant.

## Results

Demographics of the study patients are shown in Table 1. There were no differences between the two groups of infants.

**Table 1:** Demographics.

	30-45s DCC (n = 187)	60-75s DC (n = 166)	P value
GA, week, Median (Range)	30.3 (24.3, 32.9)	30.6 (24, 32.9)	0.6
BW, gram, Median (Range)	1428 (450, 2616)	1395 (430, 2530)	0.8
Antenatal Steroids, %	97	98	0.8
Antenatal Steroids >48h, %	67	68	0.8
Cesarean Section, %	61	62	0.8
Male Gender, %	65	57	0.2

GA = gestational age, BW = birth weight, DCC = delayed cord clamping.

The hematocrit values of the two DCC groups are shown in Table 2. The 60-75s DCC group had higher hematocrits at <2h compared to the 30-45s DCC group. In infants <28 weeks GA, the 12-36h hematocrit was higher in the 60-75s DCC group compared to the 30-45s DCC group.

**Table 2:** Hematocrits.

	<32weeks GA			<28 weeks GA		
	30-45s DCC (n = 187)	60-75s DCC (n = 166)	P value	30-45s DCC (n = 53)	60-75s DCC (n = 43)	P value
**<2h Hct, %, Mean (SD)** *	47.4 (7.0)	49.2 (7.0)	**0.02**	43.0 (6.1)	43.9 (6.1)	0.5
**12-36h Hct, %, Mean (SD)** **	49.8 (8.8)	51.2 (7.8)	0.1	42.1 (7.3)	47.9 (8.8)	**0.002**

DCC = delayed cord clamping, Hct = hematocrit, SD = standard deviation

* <2h hct was available for 93% of the <32weeks and 96% of the <28weeks GA.

** 12-36h hct was available for 80% of the <32weeks and 87% of the <28weeks GA.

**Fig 1:**
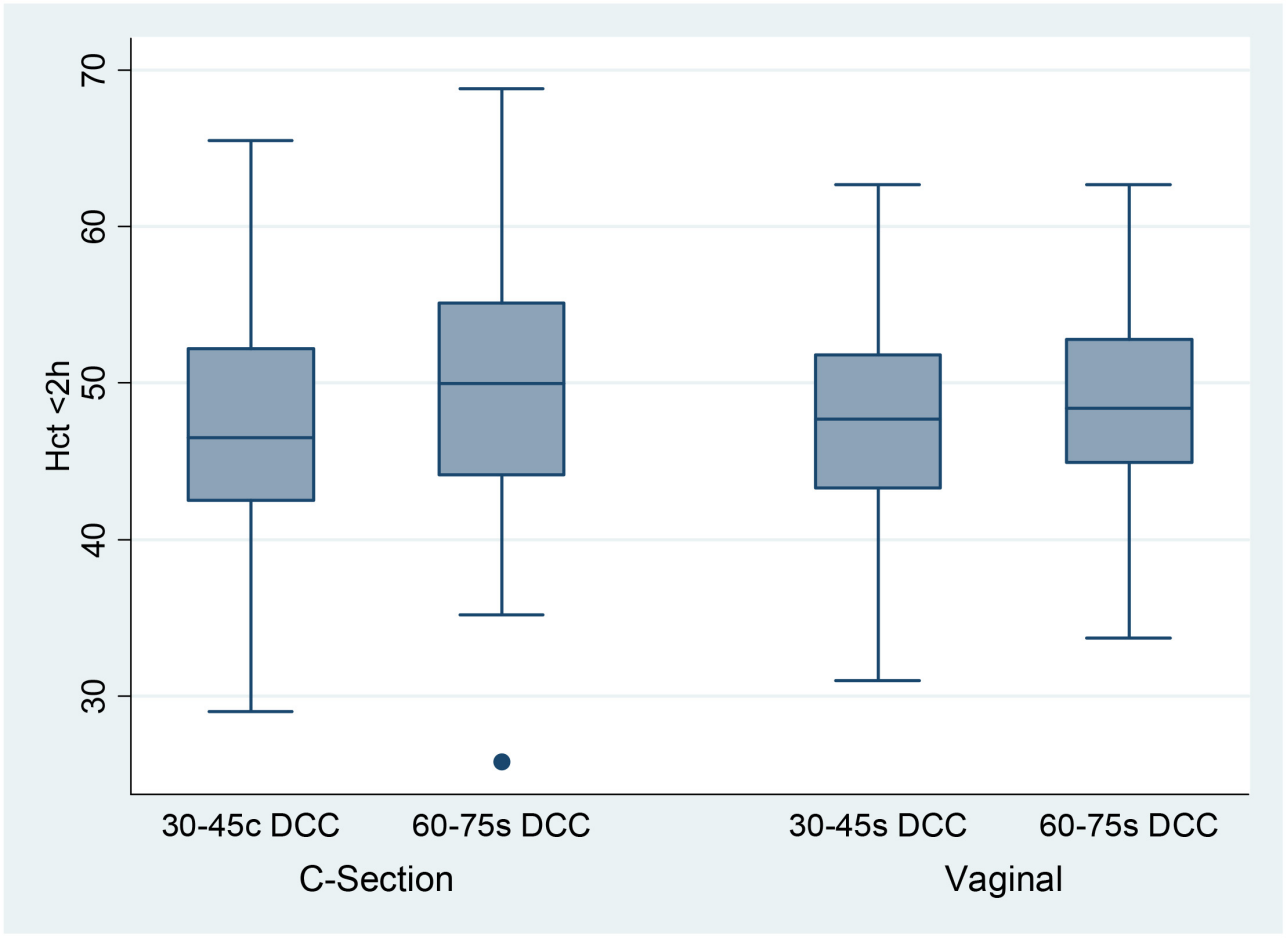
Distribution of <2h Hematocrit Values in Vaginal vs. C-Section Deliveries. The Box plots show the distribution of the hematocrit values in each group. The boxes represent the inter quartile range from 25^th^– 75^th^ percentile (IQR). The marker within the box is the median and the “whiskers” reach 1.5 times IQR.

**Fig 2:**
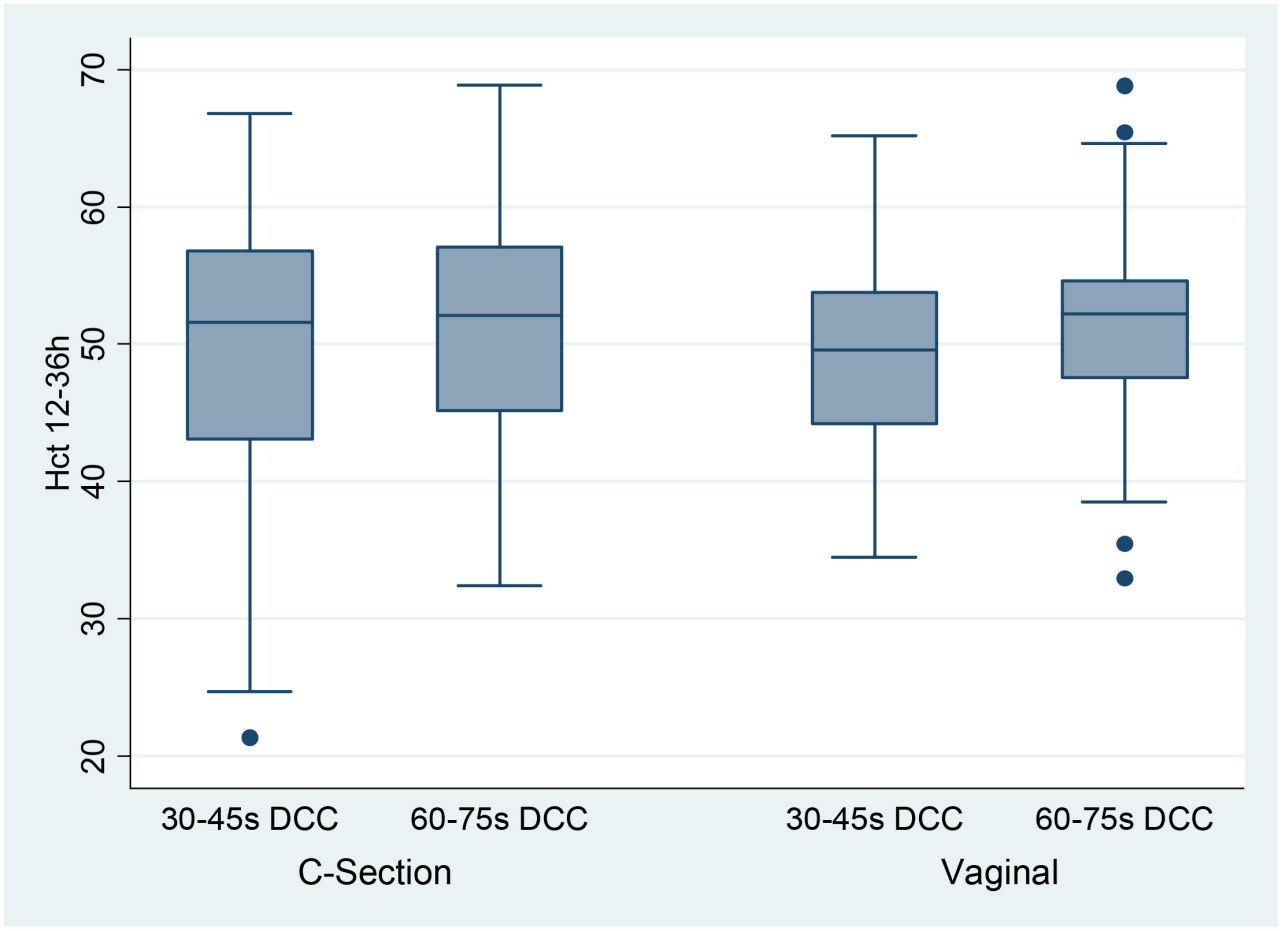
Distribution of 12-36h Hematocrit Values in Vaginal vs. C-Section Deliveries. The Box plots show the distribution of the hematocrit values in each group. The boxes represent the inter quartile range from 25^th^– 75^th^ percentile (IQR). The marker within the box is the median and the “whiskers” reach 1.5 times IQR.

Figs 1 and 2 shows the hematocrit values in CS and vaginal delivery.

DR and NICU measures are shown in Table 3. The 60-75s DCC group compared to the 30-45s DCC group had a significant reduction in hypothermia on admission, respiratory interventions (DR intubation, surfactant therapy, intubation in the first 24 hours of life and any intubation during the NICU stay), and any red blood cell transfusion during the NICU stay. Similar reductions were also observed in the <28 weeks GA subgroup except for hypothermia on admission. There were no differences in other DR and NICU measures between the two DCC groups.

**Table 3:** Delivery Room and NICU Measures.

	<32weeks GA			<28 weeks GA		
	30-45s DCC (n = 187)	60-75s DCC (n = 166)	P value	30-45s DCC (n = 53)	60-75s DCC (n = 43)	P value
**Delivery Room Measures**						
1 min Apgar, Median (Range)	6 (0,9)	7 (1,9)	0.3	5 (0,9)	5 (1,9)	0.4
5 min Apgar, Median (Range)	8 (1,10)	8 (2,9)	0.8	7 (1,9)	7 (2,9)	0.9
DR Intubation, %	22	11	**0.004**	55	33	**0.03**
DR Chest Compression and Medications, %	4	3	0.6	8	7	1.0
Admission Temperature <36°C, %	5	1	**0.01**	2	2	1.0
**Respiratory Measures**						
Surfactant, %	28	13	**0.001**	70	35	**0.001**
Intubation <24hr, %	34	20	**0.004**	77	53	**0.01**
Any Intubation *, %	40	27	**0.007**	89	63	**0.003**
Pneumothorax, %	5	2	0.3	15	7	0.3
**Hematological Measures**						
Hematocrit >65%, %	4	4	0.8	2	0	1.0
Peak Bilirubin (mg/dL),Mean (SD)	8.3 (2.6)	8.5 (2.6)	0.5	6.1 (1.8)	6.1 (1.7)	1.0
Any Red Blood Cell Transfusion*, %	33	20	**0.008**	85	67	**0.04**

DCC = delayed cord clamping, DR = delivery room, GA = gestational age, SD = standard deviation

* Defined as at least once during NICU stay.

Neonatal mortality and morbidities are shown in Table 4. There were no differences between the two DCC groups in neonatal death, any IVH, severe IVH, CLD, LOS, NEC, severe ROP or survival without major morbidities. In the <28 weeks GA sub group, any IVH was lower in the 60-75s DCC group.

**Table 4:** Neonatal Mortality and Morbidities.

	<32weeks GA			<28 weeks GA		
	30-45s DCC (n = 187)	60-75s DCC (n = 166)	P value	30-45s DCC (n = 53)	60-75s DCC (n = 43)	P value
**Death, %**	5	5	1.0	15	12	0.8
**Survival without major morbidity** * **, %**	75	73	0.8	30	33	0.8
**Any IVH, %**	24	16	0.06	57	35	**0.03**
**Severe IVH, %**	7	6	0.7	23	21	0.8
**CLD, %**	12	14	0.7	44	45	1.0
**Late Onset Sepsis, %**	8	4	0.1	21	14	0.4
**NEC, %**	5	3	0.3	11	12	1.0
**Severe ROP, %**	6	3	0.1	24	8	0.08

IVH = Intraventricular hemorrhage, CLD = chronic lung disease, NEC = necrotizing enterocolitis, ROP = retinopathy of prematurity

* Survival without major morbidities is defined as survival without severe IVH, NEC, CLD, Late Onset Sepsis, or severe ROP.

Table 5 shows multivariate logistic regression analysis. The observed reductions in hypothermia on admission, surfactant therapy, intubation in the first 24 hours, and any intubation during the NICU stay in the 60-75s DCC remained statistically significant after adjusting for GA, gender and >48 hours of antenatal steroid exposure in the entire cohort and <28 weeks GA subgroup. The reduction in any transfusion and DR intubation after risk adjusting remained significant for the entire cohort but not in the <28 weeks GA subgroup. The reduction in any IVH in the <28 weeks GA subgroup after risk adjusting was not significant.

**Table 5:** Multivariate Logistic Regression Analysis of Neonatal Outcomes Adjusted for Gestational Age, Gender and Completed Course of Antenatal Steroids.

	<32 weeks GA			<28 weeks GA		
	Adjusted* Odds Ratio	95% CI	P value	Adjusted* Odds Ratio	95% CI	P value
**DR Intubation**	0.4	0.17, 0.74	**0.006**	0.4	0.14, 1.05	0.06
**Hypothermia** **	0.1	0.01, 0.84	**0.03**			
**Surfactant**	0.3	0.16, 0.63	**0.001**	0.2	0.07, 0.52	**0.001**
**Intubation** <**24h**	0.4	0.22, 0.75	**0.004**	0.3	0.11, 0.88	**0.03**
**Any Intubation** ***	0.5	0.25,0.81	**0.007**	0.1	0.03,0.50	**0.004**
**Any red blood cell Transfusion** ***	0.3	0.17, 0.71	**0.004**	0.4	0.13,1.3	0.1
**Any IVH**	0.7	0.36, 1.23	0.2	0.5	0.21, 1.2	0.1

DR = Delivery room

*Odds ratio adjusted for gestational age, gender and >48 hours of antenatal steroids,

** Adjusted only for GA for the study cohort and no adjustment was done in the <28 weeks GA due to very low number,

*** Defined as at least once during NICU stay.

## Discussion

The benefits of DCC in preterm birth have been demonstrated in multiple clinical trials. However, there is no published study that compares the impact of different durations of DCC on DR and neonatal outcomes in very preterm infants. Our study showed that extending the duration of DCC from 30-45s to 60-75s might enhance placental transfusion benefit. Infants who received 60-75s DCC had lower hypothermia and required fewer respiratory interventions and red cell transfusions. Longer DCC did not have a negative impact on neonatal mortality and morbidity.

Earlier studies in term infants delivered vaginally showed that placental transfusion continues up to 180s after birth and that the infant’s blood volume is positively correlated with duration of DCC. Compared to 5s ICC, 30s, 60s, 90s and 180s DCC led to an increase in infant blood volume by 8.5%, 19.3%, 21.6% and 32%, respectively [4]. The physiology of placental transfusion in very premature infants is less well studied. Published studies of cord clamping in preterm infants were designed to compare placental transfusion of DCC to ICC. Data on different durations of DCC is very limited. A recent meta-analysis in preterm infants [5] showed that 30-180s DCC, compared to ICC, was associated with higher hematocrits obtained at <1 hour (3.26%), 4 hours (5.4%) and 24 hours (3.28%) of life. We observed that extending DCC from 30-45s to 60-75s led to increases in hematocrit by 1.8% at <2h of life in the whole cohort and 5.8% increase at 12–36 h in the <28 GA group. Aladangady et al [3] reported that 30-90s DCC significantly increased blood volume in very preterm infants with more pronounced increase in hematocrit and blood volume in infants with younger GA, indicating that placental transfusion may take longer to complete in these infants as there is a larger blood volume distributed in the placenta at a younger gestation. More studies are required to identify GA dependent optimal duration of DCC.

Over two thirds of very preterm infants are born by CS. Effective placental transfusion has been demonstrated in preterm infants born by CS with 30-45s DCC [8, 21]. However, concerns remain whether longer DCC in CS would cause reverse placental transfusion as previously reported in term deliveries [22, 23]. Strauss et al [2] showed that 60s DCC did increase the mean measured red blood cell volume of preterm infants delivered by CS. Aladangady et al [3], who initiated resuscitation before the cord was clamped, reported that 30–90s DCC increased blood volumes of preterm infants delivered by CS, but less than the increase observed in the vaginal deliveries. In our study, where stimulation was initiated prior to cord clamping to facilitate the onset of respiration, an additional ~30s of DCC resulted in a comparable hematocrit increase in both CS and vaginal deliveries.

Longer DCC needs to be carefully incorporated into standardized DR management in very preterm infant delivery. Our study showed that there was less hypothermia and fewer DR intubations in the 60-75s DCC group. We did not observe any difference in the Apgars, chest compressions, or epinephrine use between the two DCC groups. Interestingly, we observed that ~30s longer DCC was associated with a 50% reduction in need for DR intubation. This may not be attributable to the moderate increase in hematocrit alone. Another possible explanation is that the further delay of cord clamping allows infants to establish respiration and pulmonary circulation while the placental circulation is still intact, reducing the need for resuscitation. Recent animal studies have shown that establishing ventilation before cord clamping resulted in a more stable cardiopulmonary transition at birth [24, 25]. A recent observational study in a cohort of very low birth weight infants [26] showed a decrease in DR supplemental oxygen, positive pressure ventilation and an overall reduction in any DR resuscitative interventions in the 45s DCC group compared to the ICC group. Infants in both our study and the aforementioned observational study had high antenatal steroid exposure and were thus better prepared to initiate effective respiration with supportive stimulation. Taken together, the results from animal experiments and observational studies in humans suggest that the onset of respiration should be taken into consideration in future studies of determining the optimal time and method (DCC vs. cord milking) of cord clamping [27, 28]. Furthermore, it is important to evaluate if providing assisted positive ventilation during DCC would be beneficial in very premature infant delivery.

Respiratory outcomes were reported by few earlier DCC studies that had lower rate of antenatal steroids than current practice and a meta-analysis [5] based on these studies did not show a clear difference between DCC and ICC in respiratory distress syndrome (RDS), severe RDS, ventilation for RDS and surfactant treatment, need for oxygen at 28 days and at 36 weeks corrected GA. A more recent study [19] compared cord milking and ICC in very preterm infants who had 100% antenatal steroid exposure. Infants receiving cord milking had fewer days on oxygen therapy and less frequent use of oxygen at 36 weeks’ corrected GA. There were no differences in surfactant treatment and days on ventilation between the groups. We observed that ~30s longer DCC was associated with less respiratory interventions, including reduction in surfactant administration and intubation within first 24h, and any intubation during the entire NICU stay. This may be attributed to optimized cardiopulmonary transition and less invasive interventions at birth. However, we did not observe a change in CLD.

We observed a 40% reduction in the need for any red blood transfusion in the overall cohort and a 21% reduction in <28weeks GA who often require multiple transfusions during NICU stay. We speculate that this reduction in transfusions may be attributable to both increased blood volume and decreased phlebotomy losses in the 60-75s DCC group infants who required less respiratory interventions.

Our study is the first to compare the effects of DCC duration on the DR and neonatal outcomes of very preterm infants. However, it has limitations as a single center observational study. The providers were not blinded to DCC treatment, which is a confounding variable. The comfort level of both pediatric and obstetric providers with DCC has changed over time. However, the rates of DCC between the 30-45s DCC and 60-75s DCC study periods (78% vs. 81%) were similar. Although we had standardized DR management, intubation and transfusion guidelines during the entire study period, our results may be confounded by unintended variation in practice over time. Therefore our findings need to be further validated.

In conclusion, our study shows that extending DCC from 30-45s to 60-75s in spontaneously breathing infants promotes further placental transfusion in very preterm infants. DCC for 60-75s reduces admission hypothermia, need for intubation, and risk of blood transfusion without increasing mortality or morbidity.
